# Target of Rapamycin Mediated Ornithine Decarboxylase Antizyme Modulate Intracellular Putrescine and Ganoderic Acid Content in Ganoderma lucidum

**DOI:** 10.1128/spectrum.01633-22

**Published:** 2022-09-20

**Authors:** Tao Wu, Jiale Xia, Feng Ge, Hao Qiu, Li Tian, Xiaotian Liu, Rui Liu, Ailiang Jiang, Jing Zhu, Liang Shi, Hanshou Yu, Mingwen Zhao, Ang Ren

**Affiliations:** a Key Laboratory of Microbiology for Agricultural Environment, Ministry of Agriculture, Department of Microbiology, College of Life Sciences, Nanjing Agricultural University, Jiangsu, People’s Republic of China; b Sanya Institute of Nanjing Agricultural University, Hainan, People’s Republic of China; c Institute of Biology, Guizhou Academy of Sciences, Guizhou, People’s Republic of China; University of Michigan

**Keywords:** *Ganoderma lucidum*, ornithine decarboxylase antizyme, putrescine, ODC, TOR, secondary metabolic

## Abstract

Putrescine (Put) has been shown to play an important regulatory role in cell growth in organisms. As the primary center regulating the homeostasis of polyamine (PA) content, ornithine decarboxylase antizyme (AZ) can regulate PA content through feedback. Nevertheless, the regulatory mechanism of Put is poorly understood in fungi. Here, our analysis showed that GlAZ had a modulate effect on intracellular Put content by interacting with ornithine decarboxylase (ODC) proteins and reducing its intracellular protein levels. In addition, GlAZ upregulated the metabolic pathway of ganoderic acid (GA) biosynthesis in Ganoderma lucidum by modulating the intracellular Put content. However, a target of rapamycin (TOR) was found to promote the accumulation of intracellular Put after the GlTOR inhibitor Rap was added exogenously, and unbiased analyses demonstrated that GlTOR may promote Put production through its inhibitory effect on the level of GlAZ protein in *GlTOR-GlAZ*-cosilenced strains. The effect of TOR on fungal secondary metabolism was further explored, and the content of GA in the *GlTOR*-silenced strain after the exogenous addition of the inhibitor Rap was significantly increased compared with that in the untreated wild-type (WT) strain. Silencing of TOR in the *GlTOR*-silenced strains caused an increase in GA content, which returned to the WT state after replenishing Put. Moreover, the content of GA in *GlTOR-GlAZ*-cosilenced strains was also not different from that in the WT strain. Consequently, these results strongly indicate that GlTOR affects *G. lucidum* GA biosynthesis via GlAZ.

**IMPORTANCE** Research on antizyme (AZ) in fungi has focused on the mechanism by which AZ inhibits ornithine decarboxylase (ODC). Moreover, there are existing reports on the regulation of AZ protein translation by TOR. However, little is known about the mechanisms that influence AZ in fungal secondary metabolism. Here, both intracellular Put content and GA biosynthesis in *G. lucidum* were shown to be regulated through protein interactions between GlAZ and GlODC. Furthermore, exploration of upstream regulators of GlAZ suggested that GlAZ was regulated by the upstream protein GlTOR, which affected intracellular Put levels and ganoderic acid (GA) biosynthesis. The results of our work contribute to the understanding of the upstream regulation of Put and provide new insights into PA regulatory systems and secondary metabolism in fungi.

## INTRODUCTION

Polyamines (PAs), including putrescine (Put), spermidine (Spd), and spermine (Spm), are multivalent small molecules with an abundant intracellular content (1). PAs can bind negatively-charged substances such as DNA, RNA, and proteins through hydrogen bonds; therefore, PAs can stabilize their structure and change their conformation (2). The first product of the PA synthesis pathway is Put, which is formed by decarboxylation of ornithine through ornithine decarboxylase (ODC) in fungi. The PA spermidine has been reported to regulate mitochondrial reactive oxygen species (ROS) homeostasis in *G. lucidum* (3). PAs indirectly activate metabolite production by stimulating different signaling pathways as a part of the stress reversal process (4). In research on cucumber, foliar application of Put can alleviate the stomatal closure and photosynthesis decline caused by salt stress and promote cucumber growth (5). At present, research on Put in cells has mainly focused on fields related to homoeostasis. It was demonstrated that bacterial Put acts as a substrate for symbiotic metabolism and is further absorbed and metabolized by the host, thereby helping to maintain mucosal homoeostasis in the intestine (6). PAs are involved in controlling the formation of biofilms in bacteria (7). Consistent with this finding, exogenous Put robustly induces biofilm formation in P. aeruginosa (8). In a previous study, Put was shown to reduce intracellular ROS levels by altering the transcription and enzyme activity levels of intracellular antioxidant enzyme systems, thus ultimately affecting the accumulation of ganoderic acid (GA) (9). Overall, ODC-mediated Put plays a defensive role in various environmental stresses, but there is less research on how Put is regulated in fungi.

Antizyme (AZ) is the major regulator of intracellular PA content. AZ was discovered and proven to be an endogenous antienzyme of ODC. Although AZ is a small molecular protein with an average molecular weight of 33 kDa, it is still a major regulator of intracellular PA content. In research on mammals, the antizyme (AZ) and antizyme inhibitor (AZI) that regulate the first enzyme (ODC) in PA biosynthesis and PA uptake activity in response to intracellular PA levels have been reviewed (10). Structural analysis has demonstrated that AZ1 shuts down PA biosynthesis by physically blocking the formation of the catalytically active ODC homodimer and by targeting ODC for ubiquitylation-independent proteolysis by exposing a cryptic proteasome-interacting surface (11). AZ acts as a regulatory hub for the homeostasis of PAs and is largely dependent on its specific translation mechanism. The frameshift of the special translation mechanism is a +1-shift mechanism induced by PAs (12). The mRNA sequence of AZ includes two partially overlapping open reading frames (ORFs), with ORF1 ending in a “UGA” stop codon, while the structural domain that actually carries out the function of the AZ protein is encoded in ORF2. The frameshift mechanism is activated when intracellular PAs reach a certain level (13). As mentioned above, the main function of AZ is to maintain the balance of intracellular PA content. Research on cells provides evidence illustrating that PAD4-mediated AZ citrullination upregulates cellular ODC and PAs by retarding ODC degradation (14). A study in Saccharomyces cerevisiae showed that synthesis of yeast AZ (Oaz1) involves polyamine-regulated frameshifting as well. Degradation of yeast ODC by the proteasome depends on Oaz1 (15). Except for functional AZ protein translation levels that are altered by regulation of intracellular PA content, the TOR located upstream of nutrient metabolism regulation can also change the protein content of AZ by affecting the phosphorylation level of its downstream ribosomal translation-related kinases. The expression of AZ protein is significantly induced in mouse embryonic fibroblasts after culture in amino acid-deficient serum (16). This TOR-regulated expression of AZ protein is based on translational shifts achieved by TOR regulation of the ribosomal translation machinery and is not dependent on the level of intracellular PAs. Taken together, AZ affects intracellular PAs by regulating ODC, and TOR regulates AZ, but there are few reports on the regulation of Put by AZ and TOR.

*G. lucidum* has been proven by modern medicine and pharmacology to have various medicinal effects, such as growth inhibition and cytotoxicity in tumor cells (17, 18). One of the active components isolated from *G. lucidum* is GA, with a high research value. The specific regulatory mechanism of its biosynthesis has become a frontier research field, with environmental factors and signaling molecules all playing an important role in the regulation of the process. Exogenous chemical induction by molecules such as salicylic acid (19), or physical induction, such as by heat stress (20), increases the intracellular level of ROS. Both of these signaling molecules can promote the transcription and the level of important genes in the GA synthesis pathway of *G. lucidum*. In contrast, the exogenous addition of hydrogen-rich water (21) and Put (9) can reduce the accumulation of GA by reducing intracellular ROS. Therefore, Put appears to be increasingly important in the study of basidiomycetes. In addition, the regulation of GA by Put may be a very interesting direction in future research.

The physiological role of AZ is not well known in fungi. In this article, the homologous *AZ* gene in *G. lucidum* was obtained by full-length cloning and designated *GlAZ*. Furthermore, the domains of AZ and the characteristics of the gene sequences were analyzed and compared. Next, protein interaction experiments were used to explore the relationship between GlAZ and ODC. To investigate the role of the *AZ* gene in mycelial growth and biomass accumulation, *AZ*-silenced strains and overexpressing strains were constructed. At the same time, the regulatory effect of the intracellular signal TOR on the *AZ* and PA systems was observed. This work will help us to reveal the mechanism of action and function of AZ, including its significance in fungal physiological activities.

## RESULTS

### Cloning and analysis of the *GlAZ* gene and the GlAZ protein.

**FIG 1:**
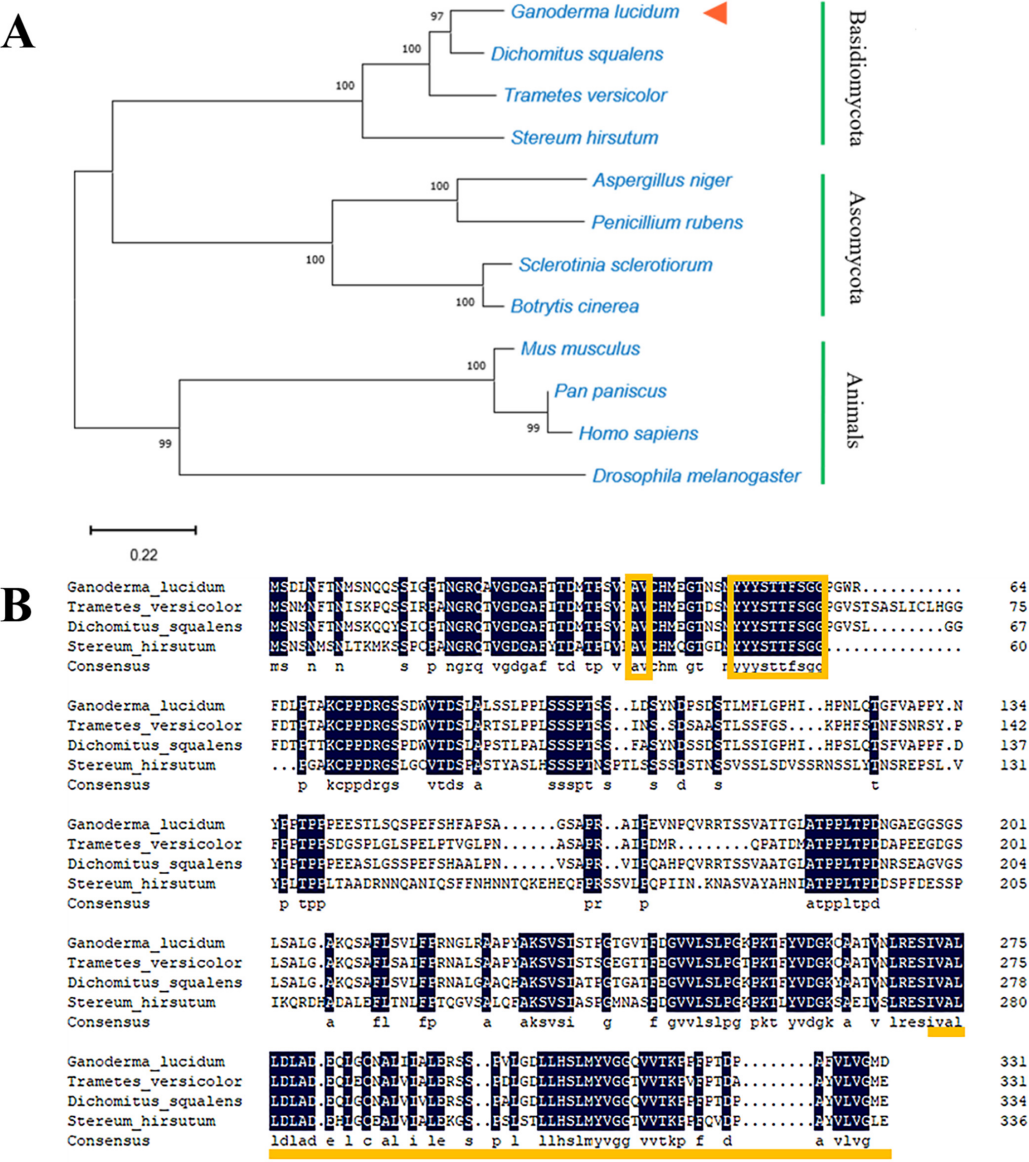
Phylogenetic analysis and alignment of the AZ domains in *G. lucidum* and other eukaryotes. (A) The evolutionary tree of GlAZ in 12 different species. (B) Alignment of AZ protein sequences of GlAZ with other basidiomycetes. The first box is conserved dipeptide structure; the second box is nascent signal peptide structure. The underlined part is the AZ functional domain; the shaded part is 100% conserved.

The frameshift translation phenomenon of AZ also exists in *G. lucidum*. The cDNA of the *GlAZ* gene is 1,000 bp with two open reading frames and a frameshift translation of the gene sequence structure “TTTGA” (Fig. S1A in the supplemental material). The entire gene of *GlAZ* encodes 66 amino acids for short-chain and 333 amino acids for full-length functionality. A total of 12 species were included in the phylogenetic tree of GlAZ: ascomycetes, basidiomycetes, and animals. The GlAZ protein is closely related to the AZ proteins of other basidiomycetes. Consequently, it naturally grouped into the same cluster. In addition, the GlAZ protein of *G. lucidum* is obviously distinct from AZ proteins of ascomycetes and animals (Fig. 1A). Expasy predicted that the molecular weight of the GlAZ protein is 34.7 kDa and that the isoelectric point is 4.82. This is similar to the molecular weight and isoelectric point of other known basidiomycete AZ proteins. After alignment with the other three basidiomycetes, the sequence alignment results showed that the similarity between them was 71.60%. A conserved dipeptide (AV), a neonatal signal peptide (YYYSTTFSGG), and a prominent AZ domain feature are present in the GlAZ amino-acid sequence (Fig. 1B).

### GlAZ inhibited mycelial growth and biomass accumulation.

**FIG 2:**
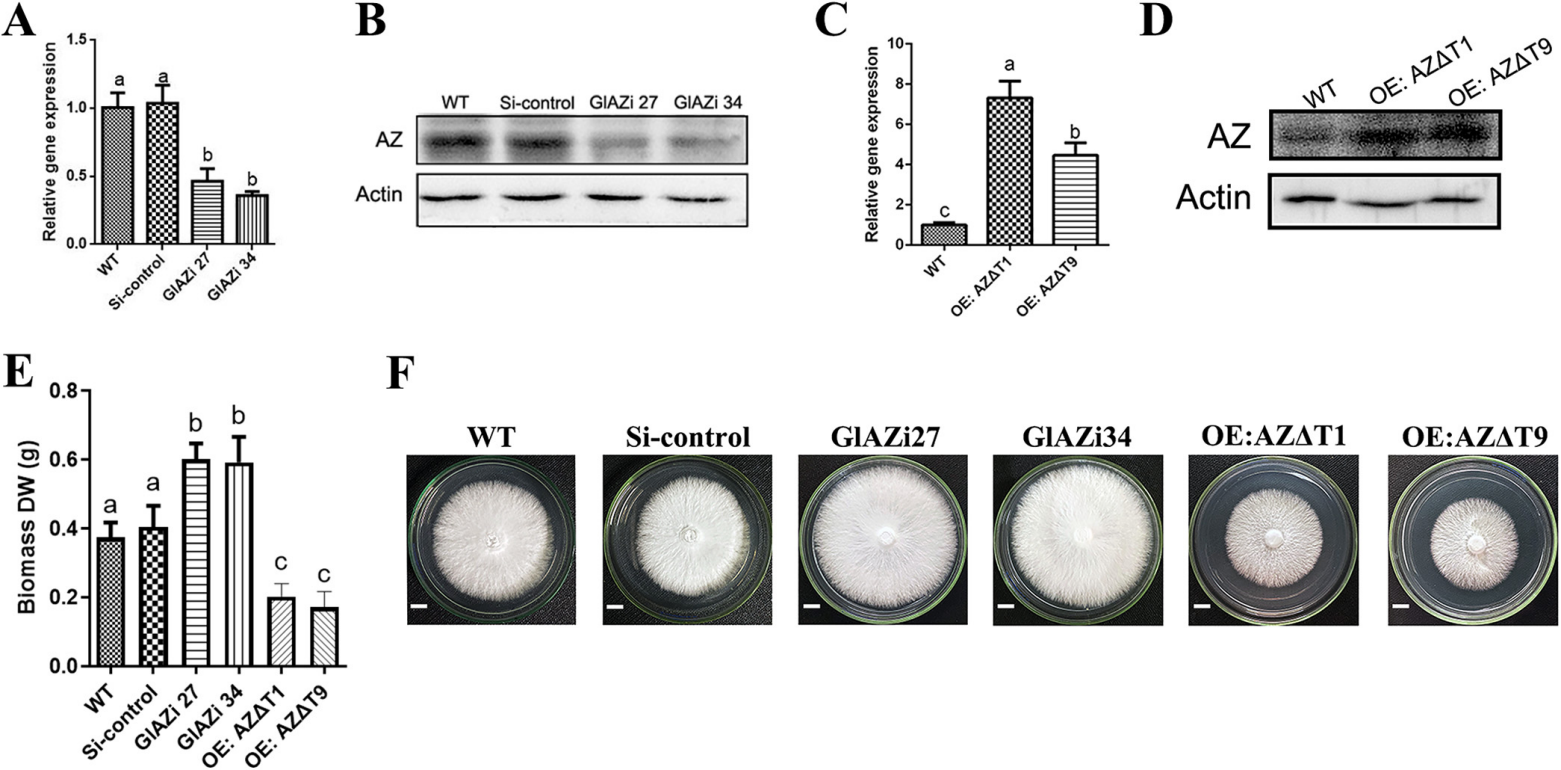
The construction of *GlAZ* mutant strains and its effect on the growth of *G. lucidum* mycelium. (A) Transcriptional levels of *GlAZ* in different strains. The *GlAZ* expression level in the WT strain was defined as 1.0. (B) Protein levels of GlAZ in different silenced strains. (C) Transcriptional levels of *GlAZ* in different strains. The *GlAZ* expression level in the WT strain was defined as 1.0. (D) Protein levels of GlAZ in different overexpressing strains. (E) Biomass dry weight statistics of mutant strains. (F) The mycelium growth of mutant strains culture in plate. Scale bar = 1 cm. Each statistical experiment was repeated at least 3 times independently. The experimental data shown in the graph are presented as the mean ± standard deviation (SD). The different letters in the graph indicate significant differences between the lines (*P* < 0.05, Duncan’s multiple range test).

A 393-bp target fragment was inserted into the original pAN7-ura30-dual plasmid for the construction of the *GlAZi* double-promoter conversion vector (Fig. S2A). Compared with the WT strain (relative expression of 1), the *AZ* transcript level in *GlAZi27* strain decreased by 54%, and those in *GlAZ34i* strain decreased by 64% (Fig. 2A). Comparing the expression level of the GlAZ protein (Fig. 2B), it was observed that the expression levels of the GlAZ protein in the *GlAZi27* and *GlAZ34i* strains were significantly lower than that in the WT strain. These findings indicate the successful construction of the *GlAZi* mutant. The *GlAZΔT* fragment was inserted into the original plasmid pGl-gpd to form a vector for overexpressing *GlAZΔT* strains (Fig. S2B). The constructed mutant strain was designated *OE:AZΔT*. Subsequently, the transcription and protein content of GlAZ in the strains were assessed using qRT–PCR and Western blotting (WB). The relative gene expression results showed that the GlAZ gene transcription levels in *OE:AZΔT1* and *OE:AZΔT9* strains were increased by 5.63-fold and 2.82-fold, respectively (Fig. 2C). Additionally, the WB findings also showed that in *OE:AZΔT1* and *OE:AZΔT9*, the levels of GlAZ protein were significantly increased (Fig. 2D). Compared with the WT strain after liquid incubation, the mycelial dry weight of *GlAZi27* and *GlAZi34* strains increased by 61% and 58%, respectively (Fig. 2E). Furthermore, the growth rates of *GlAZi27* and *GlAZi34* strains were significantly greater than those of the WT strain and the Si-control strain as determined by the diameter of the mycelium in the plate culture (Fig. 2F). In contrast, the mycelial dry weights of *OE:AZΔT1* and *OE:AZΔT9* strains decreased by 46% and 54% compared with the WT strain, respectively (Fig. 2E). Not only did the mycelial dry weight decrease, but growth significantly slowed (Fig. 2F). All these results show that GlAZ has obvious effects on the growth and biomass accumulation of *G. lucidum* and modulates its primary metabolism.

### GlAZ reduces the protein expression level of intracellular GlODC.

**FIG 3:**
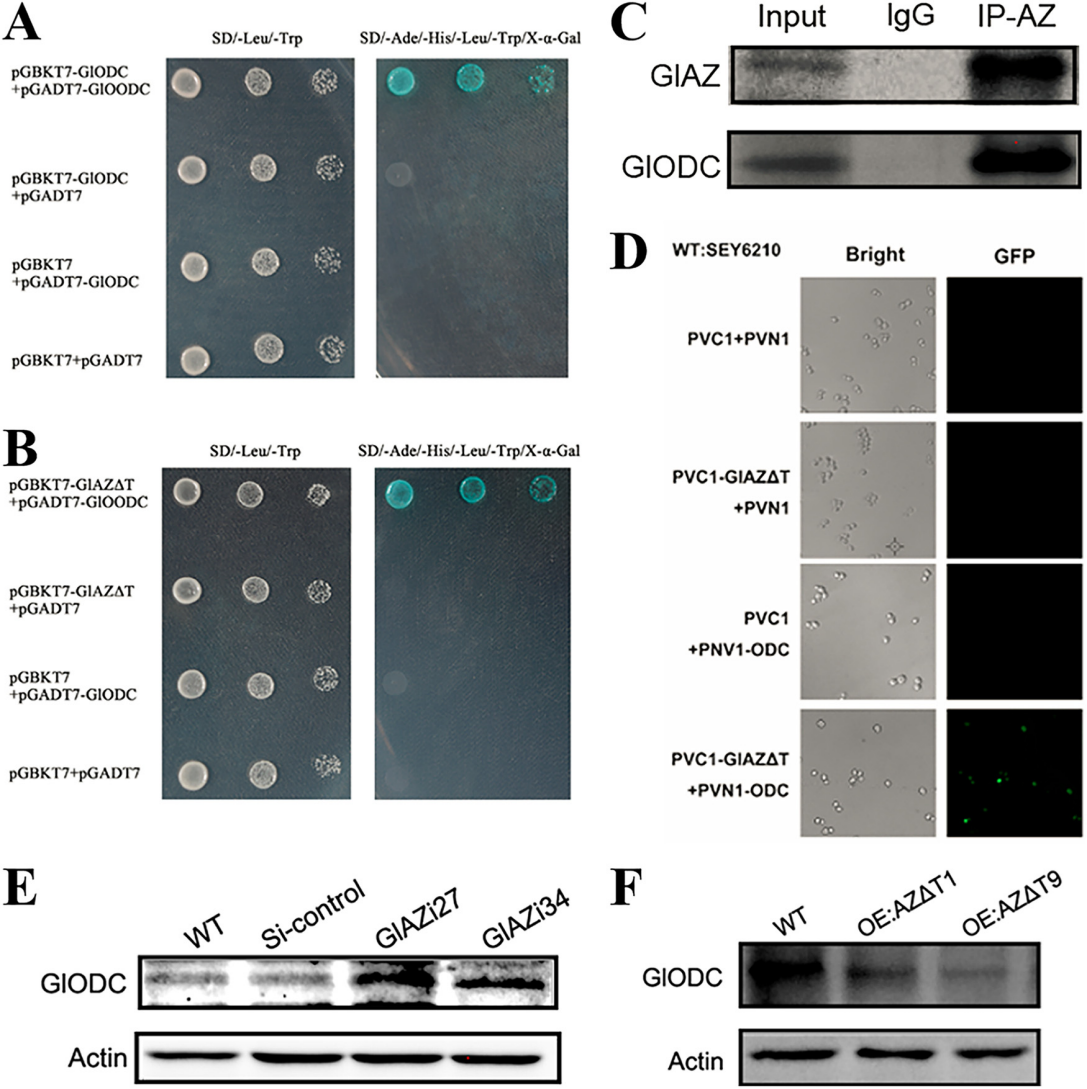
Verification of the interaction between GlAZ and GlODC protein. (A and B) Yeast double-hybrid results verification: mating formulations were screened in double deficiency medium (SD/-Leu/-Trp). (C) Co-IP for interaction between endogenous GlAZ and GlODC. (D) The result of BIFC. (E) The GlODC protein levels in *GlAZi* mutant strains. (F) The GlODC protein levels in *OE:AZΔT* mutant strains.

GlODC is a homodimer that exerts enzymatic activity as a homodimer (11). The GlODC protein subunits have been shown to interact to form a homodimer (Fig. 3A). The colonies turned distinctly blue on square-plate medium containing X-α-gal (Fig. 3B), indicating that the GlAZ protein interacted with the GlODC monomer. The WB results showed that after the specific binding of the GlAZ antibody, the GlAZ protein and the GlODC monomer were present in the protein complex (Fig. 3C). The PVN-GlAZΔT and PVC-GlODC strains produced an obvious green fluorescence reaction. In contrast, the fluorescent signal did not appear in the other controls (Fig. 3D). The results of these series of experiments verify that the GlAZ protein in *G. lucidum* can interact with the GlODC monomer. The GlODC protein levels were assessed in the *GlAZi* and *OE:AZΔT* strains. The GlODC protein levels in *GlAZi27* and *GlAZi34* were significantly higher than those in the WT and Si-control strains (Fig. 3E). Comparing the GlODC protein levels in the *OE:AZΔT1* and *OE:AZΔT9* with WT strains, a significant reduction in these protein levels was found in the mutant strains (Fig. 3F). However, the RT-qPCR data for *GlODC* demonstrated that transcription did not have a significant effect on GlODC protein levels among these strains (Fig. S3). The above research results show that GlAZ of *G. lucidum* has a significant inhibitory effect on the GlODC protein level.

### GlAZ regulates GA synthesis via GlODC and regulates intracellular Put content.

**FIG 4:**
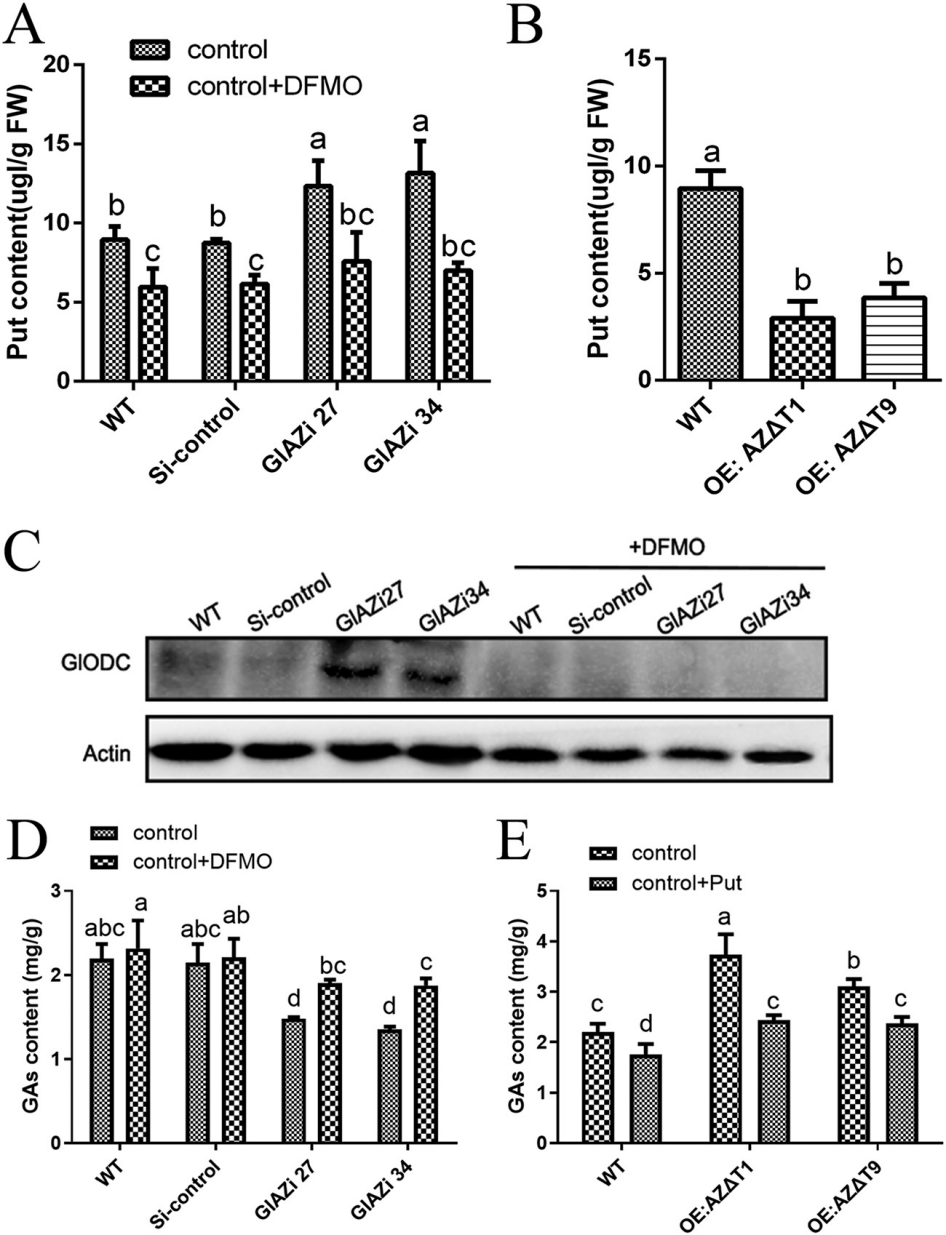
GlAZ affects GlODC/Put to regulated GA level. (A and B) The Put content in different strains. (C) WB detected GlODC protein levels. (D and E) Detection of GA content in strains. Each statistical experiment was repeated at least 3 times independently. The experimental data shown in the graph are presented as the mean ± standard deviation (SD). The different letters in the graph indicate significant differences between the lines (*P* < 0.05, Duncan’s multiple range test).

Compared with the WT strain, intracellular Put was increased by 38% and 47% in *GlAZi27* and *GlAZi34*, respectively (Fig. 4A). However, the Put content was decreased by 68% and 57% in *OE:AZΔT1* and *OE:AZΔT9*, respectively (Fig. 4B). This suggested that changes in the intracellular GlAZ protein levels can cause changes in intracellular Put content. It has also been reported that GIODC alters its intracellular protein levels (9). Difluoromethylornithine (DFMO) is a protease inhibitor of the ODC protein. DFMO was incubated with the WT, Si-control, and *GlAZ*-silenced strains. The increase in ODC protein expression levels initially due to *GlAZ* silencing was significantly decreased by the addition of DFMO (Fig. 4C). This result shows that DFMO has an inhibitory effect on the GlODC protein in *G. lucidum*. Then, DFMO was added exogenously to assess the effect on intracellular Put. The Put content, which was originally increased due to *GlAZ* silencing, showed a significant decrease after the addition of DFMO, approaching the Put levels of the WT strain (Fig. 4A). This phenotype suggests that GlAZ acts as a regulator of Put content through GlODC. The results showed that the GA levels in *GlAZi27* and *GlAZi34* decreased by 33% and 38%, respectively, compared with the WT strain. The level of intracellular GAs increased by 28% and 38% in *GlAZi27* and *GlAZi34* after exogenous addition of the GlODC inhibitor DFMO compared to that in the original silenced strains (Fig. 4D) but still did not fully revert to the level in the WT strain. The level of GAs in the *OE:AZΔT1* strain was 70% higher than that in the WT strain, while the level of GAs in *OE:AZΔT9* was also 41% higher than that in the WT strain (Fig. 4E). The GA content was assessed after exogenously supplementing the overexpression mutant strains with 1 mM Put. The results demonstrated that compared with the original untreated overexpression mutant strains, the GA content in the overexpression mutant strains decreased by 35% and 24% in *OE:AZΔT1* and *OE:AZΔT9* after Put supplementation, respectively. Summarizing these results, it is clear that GlAZ can promote the accumulation of intracellular secondary metabolites of GA by inhibiting GlODC and reducing Put content.

### GlTOR inhibits the protein expression of GlAZ via S6K.

**FIG 5:**
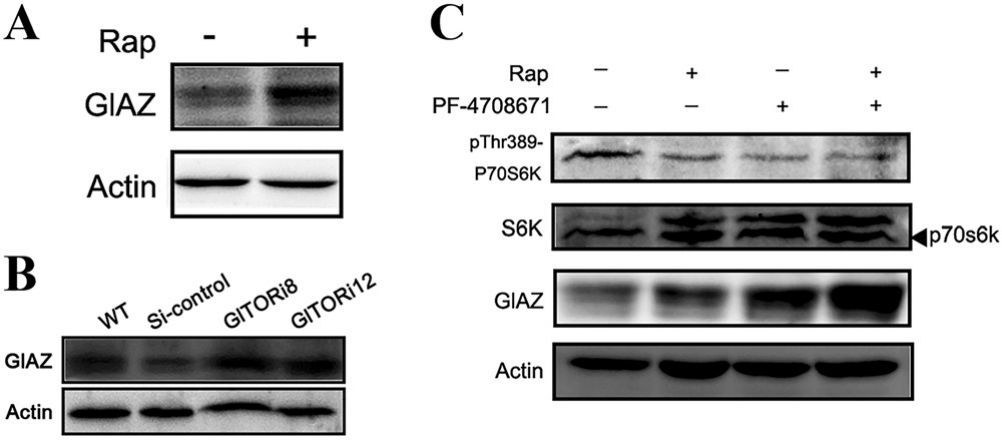
Effect of GlTOR on GlAZ detected by WB. (A, B, and C) The intracellular GlAZ protein levels were detected by WB.

Rapamycin (Rap), an inhibitor of TOR, is a lipophilic macrolide. The WT strain treated with the exogenously added Rap showed a significant increase in intracellular GlAZ protein compared to the untreated WT strain (Fig. 5A). This result was further validated by assessing *GlTORi8* and *GlTORi12*, which were constructed and preserved in the laboratory (22). Compared to the WT and Si-control strains, the *GlTORi8* and *GlTORi12* strains had more pronounced GlAZ protein bands (Fig. 5B). This result suggests that GlTOR is an inhibitor of the GlAZ protein. The PF-4708671 is a cell-permeable inhibitor of S6K; there was no significant change in the level of GlS6K protein compared to the untreated WT strain after Rap and PF-4708671 treatments. However, the degree of phosphorylation was significantly reduced. In addition, the protein phosphorylation level after PF-4708671 treatment was lower than that after Rap treatment. The level of GlS6K protein did not change significantly after cotreatment with both inhibitors. In addition, the changes in phosphorylation levels were basically consistent with the levels after PF-4708671 treatment (Fig. 5C). This indicated that both Rap and PF-4708671 could play a role in inhibiting the phosphorylation level of GlS6K. After Rap treatment, the level of GlAZ protein expression was more pronounced in the PF-4708671-treated WT strain than in the Rap-treated strain (Fig. 5C). After combined treatment with two inhibitors, the change in the GlAZ protein band was more obvious than that after treatment with a single inhibitor. However, the RT-qPCR data showed that GlTOR and GlS6K had no significant effect on the transcription level of *GlAZ* (Fig. S4). These suggest that GlTOR may inhibit GlAZ protein translation through GlS6K.

### GlTOR regulates intracellular GA content by regulating Put levels through GlAZ.

**FIG 6:**
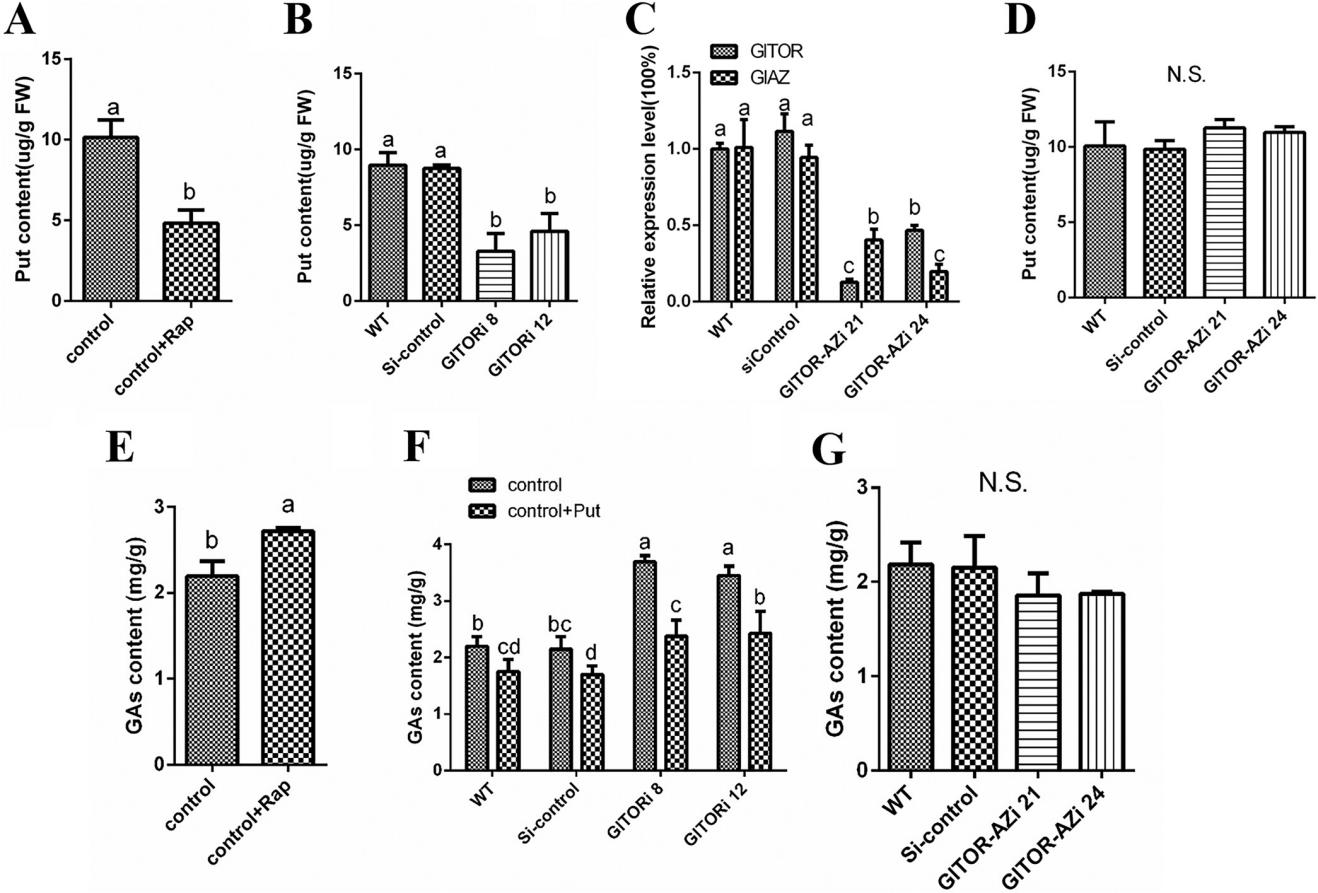
GlTOR regulates GlAZ to affect Put levels and GA content. (A and B) The Put content in different strains. (C) Transcriptional levels of *GlAZ* and *GlTOR* in different strains. The *GlAZ* and *GlTOR* expression levels in the WT strain were defined as 1.0. (D) Put content in different strains. (E, F, and G) Detection of GA content in different strains. Each statistical experiment was repeated at least 3 times independently. The experimental data shown in the graph are presented as the mean ± standard deviation (SD). The different letters in the graph indicate significant differences between the lines (*P* < 0.05, Duncan’s multiple range test). N.S., not significant.

High-performance liquid chromatography (HPLC) was used to assess the intracellular Put content, which demonstrated that the intracellular Put content of the WT strain treated with exogenous Rap decreased by 54% compared with that of the untreated strain (Fig. 6A). Compared with the WT and control strains, the Put content in *GlTORi8* and *GlTORi12* strains decreased by 63% and 49%, respectively (Fig. 6B). This finding indicates that GlTOR can promote the accumulation of intracellular Put. Remarkably, the transcript level of GlTOR was decreased by 87% in *GlTOR-GlAZi21* compared with that in the WT strain, while the transcript level of GlAZ decreased by 60% (Fig. 6C). The Put content of the cosilenced strains was assessed, and it was observed that the Put content of *GlTOR*-*GlAZi21* and *GlTOR*-*GlAZi24* strains was close to that of the WT and control strains (Fig. 6D). The above results prove that GlAZ is inhibited by GlTOR, which also indirectly promotes the increase in intracellular Put content. To explore whether TOR could regulate GAs, GA levels in the WT strain treated with Rap were increased by 23% compared with those in the untreated strain (Fig. 6E). However, the GA contents in *GLTORi8* and *GLTORi12* were 68% and 57% higher than those in the WT and Si-control strains, respectively (Fig. 6F). This finding indicates that GlTOR also plays a role in regulating secondary metabolism in *G. lucidum*. Then, Put was complemented in the *GlTORi* mutant strains. The addition of Put restored the GA levels in the *GlTORi* mutant strains to the WT strain level (Fig. 6F). However, there was no significant difference in GA content between the *GlTOR-GlAZ* cosilenced strains and the WT strain (Fig. 6G). Hence, GlTOR has an inhibitory effect on GA biosynthesis. Furthermore, GlTOR may have altered Put levels through GlAZ to reduce GA content.

## DISCUSSION

The balance of the intracellular content of PAs is important because the alteration of intracellular PAs is importantly linked to cell growth and proliferation. Knocking out ODCs in Tapesia yallundae resulted in altered intracellular PA content, which resulted in slimmer mycelium, less blackening, and sparse growth. This suggests that PAs are required in *Tapesia yallundae* to stabilize cellular components and promote normal growth (23). The induction of cystathionine γ-lyase resulted in dysregulation of the metabolism of PAs, which in turn dampened the proinflammatory response of macrophages (24). Indeed, Put is produced by ODC, which is a key role of PAs. Put-treated Psidium guajava L. exhibited a reduction in catalase and peroxidase activities (25). In G. lucidum, ODC and the ODC-mediated production of Put have been shown to influence the biosynthesis of GA (9). Put influenced GA biosynthesis by regulating NO content, possibly through nitrate reductase, under heat stress (HS) (26). Moreover, HS was shown to induce PA biosynthesis and promote the conversion of Put to Spd (27). Under iron starvation conditions, cells allocate more Put for siderophore biosynthesis by downregulating the expression of the enzyme that transforms Put into Spd (28). Subsequent research has indicated that Spd maintains mitochondrial ROS homeostasis via eIF5A hypusination, which contributes to GA biosynthesis (3). These reports indicate that the regulation of PAs and Put is fairly important in the response to stress, affecting cell growth and secondary metabolism. Accordingly, it is urgent to learn how intracellular Put is precisely regulated in *G. lucidum* to understand the regulatory role of Put.

AZ acts as an important “regulator” of the PA system. The reduction in AZ protein content reduced the inhibition of ODC by AZ at low Put concentrations; increased AZ protein content reduces ODC activity by binding to the monomer of ODC at high Put concentrations (29). Similarly, the existence of protein interactions between GlAZ and GlODC in *G. lucidum* was demonstrated in this finding. Furthermore, DFMO (an ODC inhibitor) has been shown to exert antihypertrophic and anti-apoptotic effects by inhibiting PA biosynthesis in experiments with rats (30). The GlODC protein expression level was found to be increased by GlAZ silencing in this work. The GlODC protein expression level was also reduced to WT strain levels after exogenous DFMO treatment. The frameshift was +1 and occurred at the codon just preceding the terminator of the initiating frame (12). As described in a previous report, changes in GlODC expression in *G. lucidum* causes changes in intracellular Put content (9). Our study was conducted to reveal that the regulation of Put content by GlAZ was achieved through GlODC. This completes the “regulatory” part of the homeostatic maintenance system of Put in *G. lucidum*.

Much less is known about the TOR in the regulation of AZ and ODC in fungi compared with the detailed understanding in animals and plants. Prior studies have confirmed the role of TOR in sensing intracellular nutrient content and amino acid levels (16). TOR is an important target that can be used to develop drugs against pathogenic fungi (31). In mammals, TOR influences protein translation mainly by phosphorylating 4EBP with S6K (32, 33). However, Put supplementation promotes the proliferation of porcine trophoblast cells, which is mediated by increasing protein synthesis through activation of mechanical targets of the rapamycin (mTOR) signaling pathway (34). In our experiments, it was found that GlTOR inhibited the translation of the GlAZ protein, possibly caused by GlS6K. Certainly, amino acid metabolism is also regulated by TOR, and it has been demonstrated that the TOR signaling pathway in Arabidopsis thaliana responds to amino acid levels by eliciting regulatory effects on respiratory energy metabolism at night (35). The regulatory role of TOR on Put and the product of the ODC protein were not further explored in previous studies. However, it was revealed that GlTOR promotes Put content by inhibiting GlAZ in our work.

GA are important secondary metabolites in *G. lucidum*. In addition, the functions of PAs are very broad and play a key role in the response and regulation of fungal growth and stress at specific stages. Mutations in the *AZ* gene have been shown to disrupt the intracellular PA system, altering cell growth and inhibiting cell reproduction. That study reported that AZ can affect growth without altering the intracellular PA content (36). In *G. lucidum*, there are few reports on the role of AZ in growth as in animals. However, the results of this study demonstrated that GlAZ inhibited the accumulation of biomass in *G. lucidum*. The effect of the Put “regulator” GlAZ on secondary metabolism was further explored. Our experiments demonstrated that GlAZ has a facilitative effect on the accumulation of GA. In addition, TOR is an upstream regulator of fungal secondary metabolism (37). Notably, GlTOR had an inhibitory effect on the anabolism of GA. Furthermore, it was observed that GlTOR may alter Put levels through GlAZ, thereby reducing GA content. The effect of TOR on primary metabolism such as cell growth, as well as the effect on secondary metabolism, reflects its totipotent type as an upstream regulator. In contrast, GlAZ was also involved in related metabolic pathways downstream of GlTOR. Therefore, GlAZ may be a regulatory mediator downstream of GlTOR and mediate the relationship between secondary and primary metabolism in fungi.

**FIG 7:**
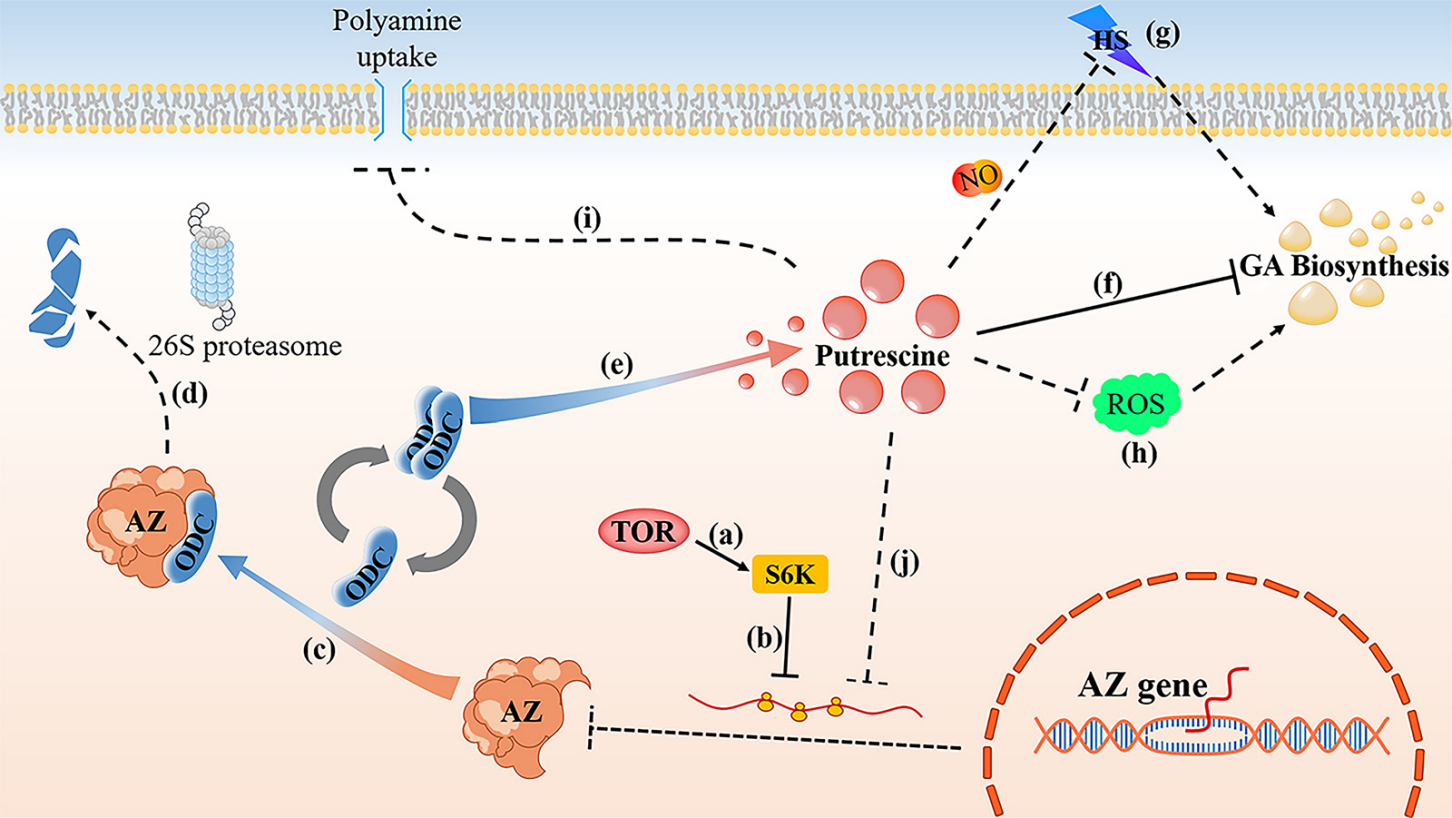
Working model of GlTOR-mediated GlAZ. (a and b) GlTOR inhibits the translation of GlAZ protein through GlS6K. (c and d) The GlAZ decrease the content of Put by both inhibiting the ODC activity and channeling ODC for proteolytic degradation by 26S proteasome. (e) The active ODC homodimer catalyzes the formation of Put from l-ornithine. (f) GA biosynthesis is inhibited by Put in *G. lucidum*. (g) Put leads to increase NO production to relieve HS-induced GA accumulation (26). (h) Meanwhile, the biosynthesis of GA by regulating the levels of ROS via Put (9). (i and j) High intracellular content of PA induces the AZ inhibition of polyamine uptake and inhibition of AZ translation (29, 46).

In summary, the work in this paper establishes a framework showing that GlTOR modulates intracellular Put and GA content through its inhibitory effect on GlAZ protein translation (Fig. 7). The GlTOR reduces the transcriptional level of GlAZ via GlS6K. Ultimately, it was demonstrated that GlAZ inhibits GlODC protein activity by binding to GlODC monomer, revealing that GlAZ causes a decrease in intracellular Put content via GlODC. These results in *G. lucidum* have helped to increase our fundamental understanding of PA regulatory systems and deepen our knowledge of the regulatory networks of fungal secondary metabolism.

## MATERIALS AND METHODS

### Fungal strains and culture conditions.

*G. lucidum* was provided by the Agricultural Culture Collection of China with the number ACCC53264. The wild-type (WT) strain was activated on potato dextrose agar (PDA) solid medium and incubated at 28°C for 7 days. The silenced strains (*GlAZi27*, *GlAZi34, OE:AZΔT1*, and *OE:AZΔT9*) were cultured at 28°C in CYM medium (2% glucose, 1% maltose, 0.05% MgSO_4_·7H_2_O, 0.2% yeast extract, 0.46% KH_2_PO_4_, and 0.2% tryptone) (38). The Escherichia coli (E. coli) DH5α strain was preserved in our laboratory. The DH5α strain was cultured in Luria-Bertani (LB).

### Gene cloning and bioinformatics sequence analysis.

The *AZ* nucleotide sequence of *Phaffia rhodozyma* (NCBI reference sequence: CED84662.1) was used as a query sequence to perform local BLAST analysis with the *G. lucidum* genome database (39), and two genes were obtained: GL25588-R1 and GL25588-R2. Through DNAMAN software comparison, it was found that there was an obvious “GT-AG” intron region in GL25588-R1. Subsequently, T-A cloning, E. coli transformation, and sequencing were performed. It was determined that there was only one *AZ* gene in *G. lucidum*, which was designated *GlAZ*. All primers were designed by Primer 5.0 software. The *G. lucidum* genomic DNA and reverse-transcribed cDNA were used as the template with the primers listed in Table S1, which were used to amplify the full-length sequence by PCR. Moreover, Expasy was used to predict the molecular weight and isoelectric point of the *GlAZ* protein. In addition, the online NCBI Conserved Domain Database and Pfam were used to predict the protein domains contained in *GlAZ*. In addition, MEGA 11 software was used to construct the *AZ* phylogenetic tree with the neighbor-joining (NJ) method. A bootstrap consensus tree with 1,000 bootstrap replications represented the evolutionary history.

### Protein expression and purification.

Translation of the protein was performed using the primers listed in Table S1 to truncate and clone the AZ domain contained in AZ. The fragment was named AZ341 due to its translation from the “ATG” of codon 341 of *GlAZ* (Fig. S1A). The amplified product was used to construct the recombinant plasmid pET-28a-AZ341 with vector pET-28a and the primers listed in Table S1. The overlap method was used for amplification, and the primers are listed in Table S1. The “T” in the stop codon of ORF1 was removed to consistently translate the full length of GlAZ (Fig. S1A and B). The target fragment was designated *AZΔT* and inserted into pET-32a to form the recombinant plasmid pET-32a-AZΔT using the primers listed in Table S1. pET-32a-AZΔT was used for the induction and expression of the full-length protein. After sequencing, the plasmid was transformed into the E. coli *BL21* expression strain for protein induction and expression. After protein induction by isopropyl-β-D-thiogalactopyranoside (IPTG) and sonication (Fig. S3), the inclusion body protein was purified with protein purification magnetic beads according to the manufacturer's instructions (Fig. S4). The purified AZ protein (concentration requirement of 0.2 mg/mL, total protein requirement of 5 mg) was subjected to low-temperature vacuum drying and sent to Shanghai Kaijing Biological Company for preparation of the rabbit immune GlAZ polyclonal antibody required for Western blotting (WB).

### Western blotting.

The mycelium samples of *G. lucidum* were ground with liquid nitrogen to extract total protein. Western blotting was performed as described previously (40). The purified GlAZ-positive protein expressed in the expression vectors pET-28a and pET-32a was used as the identification control. An anti-GlAZ antibody (1:1,000, rabbit polyclonal) was used as a primary antibody to detect the specific proteins.

### Real-time PCR analysis of gene expression.

Total RNA was extracted from *G. lucidum* hyphae using RNAiso Plus (TaKaRa, Dalian, China) as described in a previous study (41). A 5× All-In-One RT MasterMix kit (TaKaRa) was used to obtain the cDNA. *G. lucidum* hyphae were ground into powder with liquid nitrogen, and DNA was extracted by the CTAB method. Quantitative real-time RT-PCR (qRT-PCR) analysis was performed using the EvaGreen 2× qPCR MasterMix kit (ABM, Zhenjiang, China) with the primers listed in Table S1. The gene fragments were amplified by real-time PCR using primers based on the *G. lucidum* genome sequence with the primers shown in Table S1. The *GlAZ* mutant strain expression was evaluated by calculating the difference between the threshold cycle (*C_T_*) value of the gene analyzed and the *C_T_* value of the housekeeping gene 18S rRNA with the primers listed in Table S1. Quantitative reverse transcription-PCR (qRT-PCR) calculations analyzing the relative gene expression level were performed according to the 2^-ΔΔCT^ method as described in a previous study (42).

### Construction of knockdown strains and overexpression strains.

Construction of *GlAZ* gene knockdown vectors and the transformation of *G. lucidum* were performed as previously described (42). The *GlAZ* coding region was amplified by PCR using *G. lucidum* cDNA as a template with the primers listed in Table S1. The amplified PCR product was T-A ligated with the pMD19-T vector (TaKaRa). The 393-bp target fragment was inserted into the pAN7-ura30-dual original plasmid to construct a transformation vector with dual promoters for silencing the *GlAZ* gene. All primers are listed in Table S1. Finally, this plasmid was used to transform the *G. lucidum* strain and designated *GlAZi*. In addition, the construction of a fungal overexpression vector has been described in a previous work (43). The *GlAZΔT* fragment was amplified by PCR with the primers listed in Table S1. Afterward, the *GlAZΔT* fragment was inserted into the original plasmid pGl-gpd (Fig. S2B) to construct a vector for overexpressing *GlAZΔT* with the primers listed in Table S1. The plasmid was transformed into the *G. lucidum* strain by Agrobacterium tumefaciens-mediated transformation (ATMT). The constructed mutant strain was designated *OE:AZΔT* (Fig. S5).

### Estimate of mycelial growth rate and biomass.

The mycelium diameter was recorded and calculated at 28°C for 5 days after inoculation on a solid plate. Uniformly broken liquid mycelia were inoculated in CYM liquid medium at a volume ratio of 1:100 on a shaking table at 28°C and 150 rpm/min. After 5 days of culture, mycelial pellets were collected and dried to determine the dry weight.

### Yeast two-hybrid assays.

The yeast two-hybrid experimental method was carried out mainly according to the instructions of the manufacturer. Yeast two-hybrid vectors pGBKT7 and pGADT7 were purchased from Clontech Biosciences (Palo Alto, CA, USA). In brief, full-length *GlAZΔT* was ligated into the pGADT7 vector to construct the capture vector with the primers listed in Table S1. The full-length GlODC was ligated into pGADT7 and pGBKT7 to construct the capture and bait vectors, respectively. All primers are listed in Table S1. The capture vector was transformed into yeast strain Y187, and the bait vector was transformed into strain Y2H gold. After a round of auxotrophic screening of Y187 and Y2H gold-positive strains, the successful mating strains were screened in double-deficiency medium (SD/-Leu/-Trp) by preliminary mating hybridization. Finally, the interaction between the proteins was verified on quadrupole-deficiency plate medium containing X-α-gal.

### Coimmunoprecipitation assays.

To further confirm the protein interaction between GlAZ and GlODC, the coimmunoprecipitation (Co-IP) technique was used to verify the protein interaction *in vivo*. The Co-IP experimental methods were mainly based on those outlined in previously published research (44). In summary, the GlAZ polyclonal antibody was used to bind to the GlAZ protein in *G. lucidum*. Subsequently, the GlAZ antibody was bound to the magnetic beads, and protein components in the antibody-bound protein complex were assessed by WB.

### Bimolecular fluorescence complementation assays.

Yeast was used as a carrier strain to carry out bimolecular fluorescence complementation (BIFC) experiments, which have matured in recent years. The experimental method was mainly based on methods outlined in previously published research (45). After hybridization in medium, the constructs PVN-GlAZΔT and PVC-GlODC were prepared into samples and observed under a fluorescence microscope.

### Quantification of intracellular Put content.

The sample preparation method for the detection of Put was performed as outlined in a previous study (26). Put was quantified using high-performance liquid chromatography (HPLC). Briefly, 3 mL of 5% (vol/vol) cold perchloric acid was added to the obtained 0.2 g mycelia powder. The mixture was transferred to a plastic tube and placed on ice for 1 h. The mixture was then centrifuged for 30 min at 12,000 g, 4°C; 2 mL of supernatant was collected, to which 1 mL of 2 M NaOH was added. The mixture was vortexed, and 10 μL of benzoyl chloride was added to the mix. The mixture was incubated in a water bath at 37°C for 30 min before adding 2 mL of saturated NaCl. After adding 2 mL of diethyl, the samples were mixed vigorously and then phase separated at 4°C and centrifuged at 3,000 × *g* for 10 min. After drying an aliquot (1 mL) of the organic solvent phase with nitrogen, the residue was resuspended in 1 mL of methanol. The analysis of benzoylated Put was performed with HPLC (i-Serise, Shimadzu, Japan) using a reversed-phase column and measured at 230 nm.

### Quantification of GA content.

The method of extracting GA was as described previously (41). GA was quantified using HPLC. Dried mycelia (0.3 g) were extracted with 10 mL of 99.5% (vol/vol) ethyl acetate for 2 h by ultrasound. After centrifugation, the supernatant was dried by rotary evaporation, and then the residue was dissolved in 1 mL of methanol. The total amount of GA was analyzed using HPLC (i-Serise, Shimadzu, Japan) equipped with a Shim-pack VP-ODS C18 column (4.6 mm × 250 mm, 5 μm). Mobile phase A contained methanol/acetic acid (1,000:1 vol/vol), and mobile phase B was 100% ultrapure water. The method employed a linear gradient from 50% A to 100% A over 20 min at a constant flow rate of 1 mL/min. The process was monitored at a wavelength of 252 nm.

### Statistical analysis.

Each statistical experiment was repeated at least 3 times independently. The experimental data shown in all graphs are presented as the mean ± SD. The experimental data were analyzed by Duncan’s multiple range test and plotted by GraphPad Prism 6. The different letters in the graph indicate significant differences between the different treatments. *P* < 0.05 was considered to be significant.

### Data availability.

The GenBank accession numbers for the various genes are shown in Table S1.
