# Supplementary material for: Target of Rapamycin Mediated Ornithine Decarboxylase Antizyme Modulate Intracellular Putrescine and Ganoderic Acid Content in Ganoderma lucidum
Source: Microbiol Spectr. 2022 Sep 20;10(5):e01633-22. doi: 10.1128/spectrum.01633-22 (PMC9604110; doi:10.1128/spectrum.01633-22)
Supplement: Supplemental file 1 — Table S1, Fig. S1 to S5. Download spectrum.01633-22-s0001.pdf, PDF file, 0.6 MB [file spectrum.01633-22-s0001.pdf]

TABLE S1 Primers used in this study

Primer	Sequence (5' to 3')	Description
RT-18S-F	TATCGAGTTCTGACTGGGTTGT	Detects the *18S* expression
RT-18S-R	ATCCGTTGCTGAAAGTTGTAT	
RT-GL25588-F	ATGTCGGACTTGAATTTTAC	Get the cDNA fragment of
RT-GL25588-R	CTAGATATCCATGCCCACGAGCACG	*G.lucidum AZ* gene
RT-GL25588-2-F	CCGAATTCATGTTTCTCGGGCCTCATATCC	Get the prokaryotic expression
RT-GL25588-2-R	TGCTCGAGGATATCCATGCCCACGAGCA	fragment of *G.lucidum AZ341* gene
RT-GL25588-3-F	CGGAATTCATGTCGGACTTGAATTTTAC	Get the prokaryotic expression
RT-GL25588-3-overlap-R	GTAGGAAGGTCAAACCGCCAGCCAGGCCCACCAGAG	fragment of *G.lucidum AZΔT* gene
RT-GL25588-3-overlap-F	CTCTGGTGGGCCTGGCTGGCGGTTTGACCTTCCTAC	
RT-GL25588-3-R	CCGCTCGAGCTAGATATCCATGCCCACGAGCACG	
RT-GL25588-4-F	CCCATATGTCGGACTTGAATTTTAC	For Yeast Two hybrid
RT-GL25588-4-R	CGGAATTCGATATCCATGCCCACGAGCACG	verification
RT-GL25588-5-F	ACTGGGTACCCCCTCCGACTCCACGCTAA	Get the silencing fragment of
RT-GL25588-5-R	ACTGACTAGTGGTGCCAGGCGTAGAAA	*G. lucidum AZ* gene
RT-GL25588-6-F	CGGGATCCATGTCGGACTTGAATTTTACCA	Get the overexpression fragment of
RT-GL25588-6-R	GCTCTAGACTAGATATCCATGCCCACGAGCACG	*G. lucidum AZΔT* gene
RT-GLAZ-F	CATTGTTGCCCTACTGGAT	Detects the *AZ* gene
RT-GLAZ-R	GGAAAGGAGGCTTGGTG	expression
RT-GLAZ-BIFC-F	CGGGATCCATGTCGGACTTGAATTTTAC	For BIFC verification
RT-GLAZ-BIFC-R	CCATCGATCTAGATATCCATGCCCACGAGCACG	
RT-ODC-F	CCCATATGGCTCAACTCGAGATCT	For Yeast Two hybrid
RT-ODC-R	CGGGATCCTCACGCGTCCAGGTGGCT	verification
RT-ODC-2-F	GCGGCTGATGTTGCGTGTC	Detects the *AZ* gene
RT-ODC-2-R	CGGCAGAGCGGATGAAG	expression

RT-ODC-BIFC-F	CGGGATCCATGGCTCAACTCGAGATCT	For BIFC verification
RT-ODC-BIFC-R	CCATCGATTCACGCGTCCAGGTGGCT	
RT-TOR-AZ-F	GGGGTACCAGGTATCAAGGGGAGCGA	Get the silencing fragment of
RT-TA-overlap-R	TCGGAGGGACAGCAAGCGAACGAGTG	*G. lucidum TOR-AZ* gene
RT-TA-overlap-F	CCCTCCGACTCCACGCTAA	
RT-TOR-AZ-R	GGACTAGTGGTGCCAGGCGTAGAAA	
RT-TOR-F	TCAACAACCTCCTTACCG	Detects the *TOR* gene
RT-TOR-R	TGACCAGACTATGCTCCC	expression

A

ATGTCGGACTTGAATTTTACCAATATGTCGAATCAACAATCTAGTATTGGCCCCACCAACGGTCGTCAAGCCGTTGGGG
ATGGGGCTTTCACCACCGACATGACTCCTAGTGTGATTGCAGTCTGCCACATGGAGGGCACGAACTCCATGTACTACTA
CTCCACCACGTTCTCTGGTGGGCCTGGCTGGCGGTTTTGACCTTCCTACTGCAAAATGCCCTCCTGACCGAGGGTCGTC
AGACTGGGTGACAGACAGTCTGGCGCTATCTTCCCTTCCCCCGCTCTCCTCTTCTCCCACCTCCTCCCTCGATTCTTACA
ACGACCCCTCCGACTCCACGCTAATGTTTCTCGGGCCTCATATCCATCCCAATCTTCAAACCGGTTTCGTCGCACCTCCT
TATAACTATCCGCCGACCCCGCCCCCGGAAGAGTCAACCCTCAGCCAATCCCCGGAGTTCTCGCACTTCGCGCCTTCCG
CTGGCAGTGCCCCGCGTGCGATCCCTGAGGTCAATCCCCAGGTCCGCCGTACGAGCTCGGTGGCTACGACCGGACTG
GCTACCCCTCCCCTGACCCCGGACAACGGAGCTGAGGGCGGTTCTGGAAGCCTCTCAGCTCTCGGTGCGAAGCAGTCC
GCATTTCTCTCTGTGCTTTTCCCCCGGAATGGCCTTCGCGCAGCTCCGTATGCAAAGAGCGTCTCAATTTCTACGCCTGG
CACCGGCGTCACGTTCGATGGTGTCGTGCTTTCACTCCCGGGCAAGCCGAAGACGTTCTACGTCGACGGCAAGTGCGC
CGCTACGGTGAATCTTCGTGAGAGCATTGTTGCCCTACTGGATCTCGCCGATGAACAGCTCGGCTGCAACGCCCTCATC
ATCGCCCTCGAACGTTCGTCGCCCGTCCTGGGCGACCTCCTCCACTCCCTCATGTACGTGGGCGGCCAGGTGGTCACC
AAGCCTCCTTTCCCCACCGACCCTGCGTTCGTGCTCGTGGGCATGGATATCTAG

B

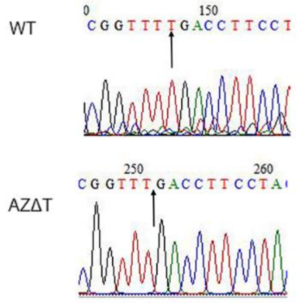

Fig.S1 *GlAZ* (GL25588) cDNA Full-Length and *GlAZΔT* Sequencing chromatogram.

(A) The ORF1 coding ends at codon 198. The gene sequence structure "TTTGA" for frameshift translation is marked in blue. The AZ341 fragment begins translation at codon 341 "ATG" is marked in green. (B) *GlAZΔT* Sequencing chromatogram.

A

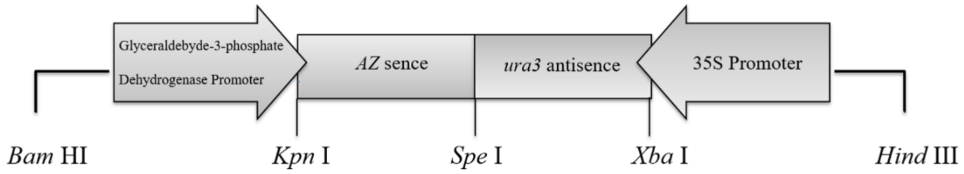

B

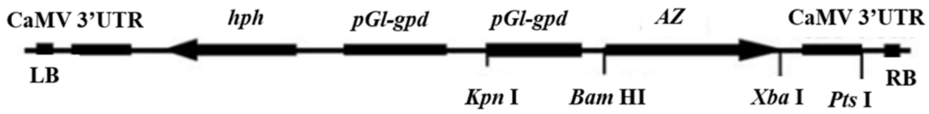

Fig.S2 Construction of *GlAZi* and *OE:AZΔT* plasmids.

(A) 393 bp target fragment was inserted into the pAN7-ura30-dual original plasmid, the transformation vector with dual promoters for the *GlAZ*-silenced gene was constructed. (B) The *gpd promoter*-*GlAZ* fusion fragment was amplified from genomic DNA.

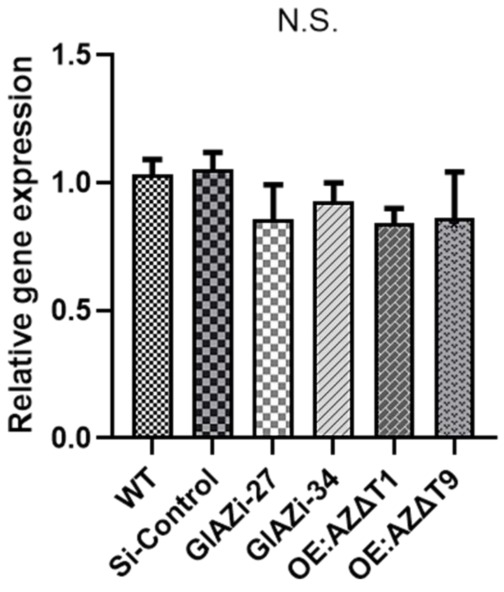

Fig.S3 Transcriptional levels of *GlODC* in different strains.

The *GlODC* expression level in the WT strain was defined as 1.0; The RT-qPCR was used to detect the transcription level of *GIODC* in these strains. N.S. is Not Significant.

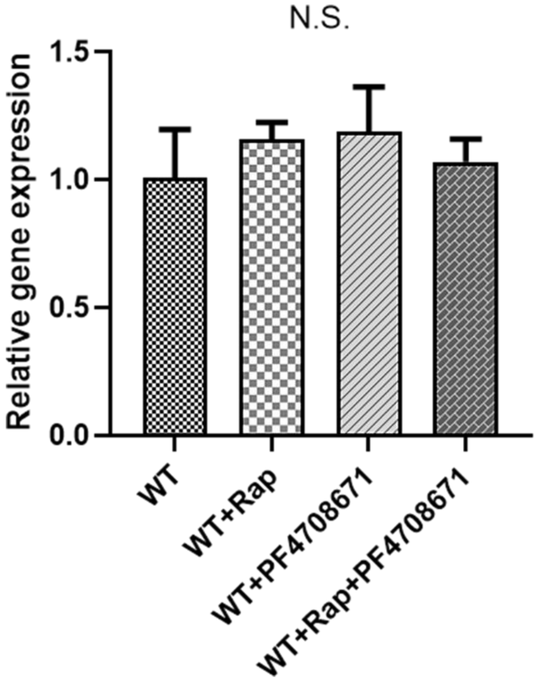

Fig.S4 Transcriptional levels of *GlAZ* in different strains.

The *GlAZ* expression level in the WT strain was defined as 1.0; The RT-qPCR was used to detect the transcription level of *GlAZ* in different treatments of WT strains. N.S. is Not Significant.

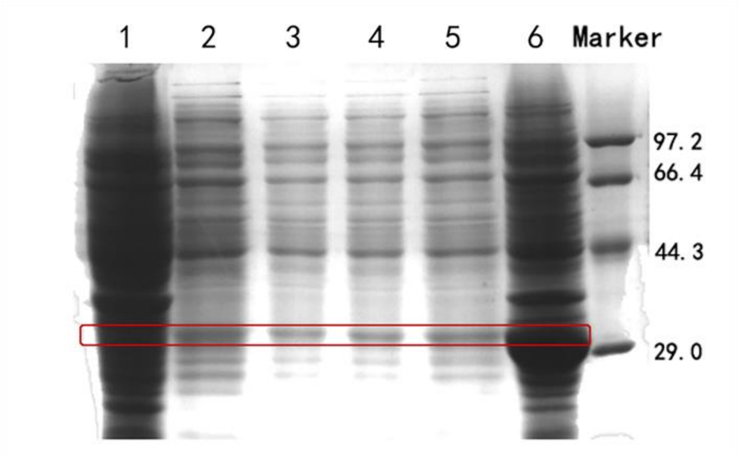

Fig.S5 Analysis of protein-induced expression by SDS-PAGE.

Small amount of induced protein expression. Lane 1: not induced; Lane 2: 0.10 mM IPTG induced supernatant; Lane 3: 0.25 mM IPTG induced supernatant; Lane 4: 0.50 mM IPTG induced supernatant; Lane 5: 1.00 mM IPTG induced supernatant; Lane 6:0.10 mM IPTG induced inclusion body.

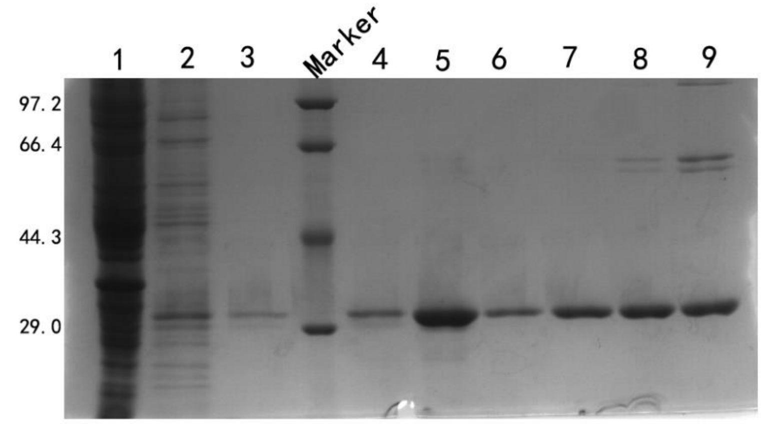

Fig.S6 SDS-PAGE analysis of protein purification.

Lane 1: uninducible protein; Lane 2: 20 mM imidazole fluid; Lane 3-9: followed by 50, 80, 100, 150, 200, 250, 400 mM imidazole eluent.

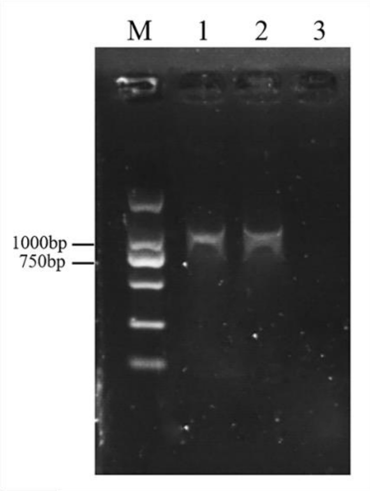

Fig.S7 The *gpd promoter-GlAZ* fusion fragment was amplified from Genomic DNA.

Lane 1 is *OE: AZΔT1*, Lane 2 is *OE: AZΔT9*, and Lane 3 is the WT strain.
